# Atrial Fibrillation and Chronic Kidney Disease: Aetiology and Management

**DOI:** 10.31083/j.rcm2504143

**Published:** 2024-04-09

**Authors:** Bharat Sidhu, Akash Mavilakandy, Katherine L. Hull, Ivelin Koev, Zakariyya Vali, James O. Burton, G. André Ng

**Affiliations:** ^1^Department of Cardiovascular Sciences, University of Leicester, LE1 7RH Leicester, UK; ^2^Department of Cardiology, University Hospitals of Leicester NHS Trust, LE1 5WW Leicester, UK; ^3^John Walls Renal Unit, Leicester General Hospital, LE5 4PW Leicester, UK; ^4^School of Sport, Exercise and Health Sciences, Loughborough University, LE11 3TU Loughborough, UK; ^5^National Institute for Health Research Leicester Biomedical Research Centre, LE3 9QP Leicester, UK

**Keywords:** atrial fibrillation, chronic kidney disease, anticoagulation

## Abstract

Chronic kidney disease (CKD) and atrial fibrillation (AF) are associated with significant cardiovascular morbidity and mortality. Recent studies have highlighted an increased prevalence and incidence of AF in patients with CKD. This article aims to provide a comprehensive review of current management strategies and considerations of treating atrial fibrillation with concomitant CKD. Potential electrophysiological mechanisms between AF and CKD are explored. Current evidence and literature focusing on pharmacological rate and rhythm control along with procedural intervention is reviewed and presented. The management of AF and CKD together is complex, but particularly pertinent when considering the close cyclical relationship in the progression of both diseases.

## 1. Introduction

Atrial Fibrillation (AF) is the most common sustained cardiac arrythmia with an estimated prevalence of 1–2% in the general population [1]. In Europe the number of adults affected by AF in 2010 was estimated to be 8.8 million (95% CI 6.5–12.3 million) which reflects 1.8% of the adult population aged ≥55 years and there are projections that by the year 2060 this will increase to approximately 17.9 million people (95% CI 13.6–23.7 million), which reflects 3.5% of the adult population [2]. The occurrence of isolated AF (termed ‘lone AF’) is rare. There is mounting evidence that lifestyle and several cardiovascular risk factors play a significant role in the initiation, progression, and maintenance of AF. Cardiovascular and lifestyle risk factor modification has been shown to improve AF outcomes [3].

Although there are several traditional cardiovascular risk factors associated with chronic kidney disease (CKD), it is important to acknowledge the role of non-traditional risk factors, such as metabolic acidosis, oxidative stress, uraemia, chronic inflammation, anaemia, disrupted mineral bone homeostasis and chronic volume overload, in individuals with advanced CKD that do not respond to current recommended risk reduction strategies [4, 5, 6]. The presence of AF and CKD provides a clinical challenge with regards to pharmacological management, anti-thrombotic therapy, and whether to pursue a rate or rhythm control strategy. Up to 30% of patients diagnosed with AF have stage III-V CKD [7].

## 2. AF and CKD: Electrophysiological Mechanisms

The initiation of AF is caused by a complex interaction between a trigger and substrate. It is the modification of the anatomical and/or electrical properties of the atria that gives rise to the underlying substrate. Cardiac chamber remodelling, in particular of the left atrium, which occurs secondary to sustained volume overload, elevated filling pressure and contractile dysfunction provides the substrate necessary for initiation, propagation and maintenance of AF [8]. Elevated atrial pressures may be found in patients with CKD due its association with hypertension. Therefore, the mechanical stress exerted on the atria, overtime, may result in electrophysiological remodelling which leads to the development of AF [9].

Overall, the development of AF in patients with CKD is multifaceted with other potentially relevant components including inflammation, renin-aldosterone-angiotensin-system (RAAS) activation, electrolyte abnormalities, anaemia and uraemia [10, 11, 12, 13].

## 3. AF and The RAAS

The RAAS is an endocrine and paracrine system which has an important role in the regulation and modulation of renal, cardiovascular and pulmonary processes [14]. The RAAS cascade is also key in the progression of CKD [15]. Studies have previously suggested the integral role of RAAS in the pathogenesis of AF. Angiotensin II (ATII) plays a key role in fibroblast proliferation, matrix protein accumulation and subsequent interstitial fibrosis [16]. Furthermore, the elevation in left ventricular end diastolic pressure (LVEDP) caused by ATII will lead to a subsequent rise in left atrial pressure, particularly in patients with hypertension and heart failure [17, 18]. The secondary effects of atrial dilatation include alteration of ion channels and shortened refractory periods. This has been demonstrated in animal studies. Ravelli *et al*. [19] published a study in 1997 which demonstrated that in animal studies, increases in atrial pressure resulted in shortening of the atrial effective refractory periods (AERPs) and thus increased the susceptibility to AF. Termination of AF was observed on relieving the atrial stretch.

**Fig. 1.**
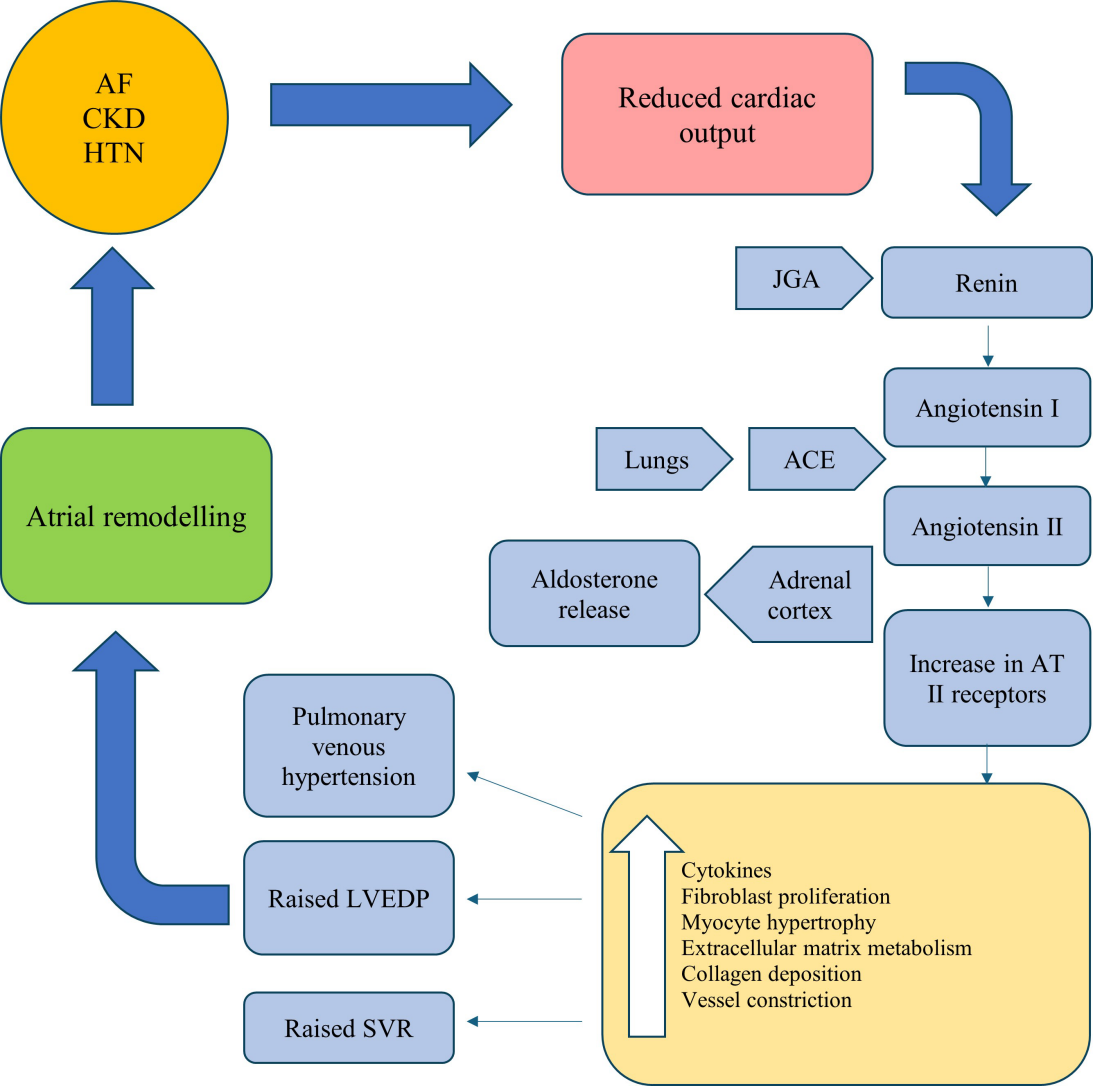
**A summary of the RAAS cascade in the pathogenesis of AF**. AF, atrial Fibrillation; CKD, chronic kidney disease; HTN, hypertension; JGA, juxtaglomerular apparatus; AT, angiotensin; ACE, angiotensin converting enzyme; LVEDP, left ventricular end diastolic pressure; SVR, systemic vascular resistance; RAAS, renin-aldosterone-angiotensin-system.

In patients with AF, heart failure and hypertension there may be prolonged activation of the RAAS, resulting in elevated myocardial tissue levels of angiotensin converting enzyme (ACE). There is a resultant up-regulation of ATII receptors which promote inflammatory response and fibrosis. The atrial remodelling that then occurs provides the substrate for sustaining AF. This cascade of events is summarised in Fig. 1 [20, 21, 22]. The atria appear to exhibit a greater susceptibility to fibrosis in comparison to the ventricles through the involvement of three interconnected pathways; RAAS, transforming growth factor β1 (TGF- β1), and oxidative stress [23]. Xiao *et al*. [24] reported a particular propensity for atrial enlargement and fibrosis in transgenic mouse models with overexpression of cardiac ACE and development of AF.

Angiotensin-converting enzyme inhibitors (ACEi) or angiotensin II receptor blockers (ARBs) are among the most established and studied antihypertensive agents that provide renal and cardiovascular benefits for CKD patients [25, 26, 27, 28]. This is likely attributed to their established efficacy in favourably modifying the structure and function of the vasculature along with the inhibition of the ATII effect of cardiac myocytes, renal glomerular pericytes, and the vascular endothelium [29, 30, 31]. ACEi have additionally shown promising application and efficacy in the management of AF potentially though favourable effects on atrial electrical, structural and functional remodelling [32, 33, 34, 35]. A prospective study conducted by Boldt *et al*. [36] reported that patients with AF that were treated with ACEi, observed an attenuation of the atrial structural remodelling along with a preservation of atrial microcapillaries. Healey *et al*. [37] conducted a systematic review and meta-analysis where they concluded that ACEi and ARBs exhibited an efficacy in AF prevention, albeit in patients with systolic left ventricular dysfunction or LV hypertrophy. Overall, the current evidence base in literature gives support to the role and benefit of ACEi in reducing the incidence of AF and severity of atrial fibrosis.

## 4. Stroke and CKD

There is a strong association between AF and stroke secondary to cerebral embolism [38]. Patients with CKD are at an increased risk of stroke and in those patients the risk of stroke is thought to be 5–30 times higher, particularly if they have end-stage kidney disease (ESKD) managed with maintenance dialysis [39, 40]. In patients with CKD, the prevalence of AF is very high when compared to the general population. In patients with an estimated glomerular filtration rate (eGFR) >45 mL/min the prevalence of AF is reported to be 16% and in those with an eGFR <45 mL/min, 20.4% [41]. The prevalence of AF is estimated to be between 3.5% to 27% depending on the type of AF, in patients on dialysis [42]. Analysis of data from the United States Renal Data System, between 1992 and 2006 showed that the one year mortality in patients on haemodialysis who had AF was two times higher than those who did not have AF (39% versus 19%) [42]. Thromboembolic events rates in patients with AF who are on haemodialysis were also observed to be 4.8-fold higher in one single centre study (24% per year in those with AF versus 5% in those with sinus rhythm) [43].

Airy *et al*. [44] concluded from their data that in non-dialysis dependent CKD concurrent AF has been associated with a higher all-cause cardiovascular mortality. Table 1 (Ref. [45, 46, 47, 48, 49]) summarises all-cause mortality reported by primary studies comparing outcomes in patients with atrial fibrillation and chronic kidney disease.

**Table 1.** **All-cause mortality reported by primary studies comparing outcomes in patients with atrial fibrillation and chronic kidney disease**.

All-cause mortality								
Study	Country	Study design	Sample size/n	Effect estimate	Comparison categories	Mean follow up/years	Results	*p* value
Hsu *et al*. [45]	Taiwan	Cohort study	16,451	HR (95% CI)	CKD: Prevalent AF vs Non-AF -Age <65	4.72 ± 3.75	1.98 (1.71–2.29)	<0.001
Hsu *et al*. [45]	Taiwan	Cohort study	16,451	HR (95% CI)	CKD: Incident AF vs Non-AF-Age <65	4.72 ± 3.75	2.07 (1.83–2.33)	0.529
Hsu *et al*. [45]	Taiwan	Cohort study	16,451	HR (95% CI)	CKD: Prevalent AF vs Non-AF-Age >65	4.72 ± 3.75	1.78 (1.68–1.88)	<0.001
Hsu *et al*. [45]	Taiwan	Cohort study	16,451	HR (95% CI)	CKD: Incident AF vs Non-AF-Age >65	4.72 ± 3.75	2.25 (2.12–2.4)	0.529
Olesen *et al*. [46]	Denmark	Retrospective study	132,372	HR (95% CI)	AF: Non-renal disease vs Non-ESRD renal disease	*	2.37 (2.3–2.44)	<0.001
Olesen *et al*. [46]	Denmark	Retrospective study	132,372	HR (95% CI)	AF: Non-renal disease vs ESRD renal disease	*	3.35 (3.13–3.58)	<0.001
Abbott *et al*. [47]	United States	Cohort study	3374	HR (95% CI)	Chronic dialysis patients: AF vs Sinus Rhythm	2.92 ± 1.14	1.54 (1.19–1.99)	<0.001
Banerjee *et al*. [48]	*	Prospective study	5912	HR (95% CI)	AF patients: eGFR 30–59 vs eGFR >60	2.45	1.98 (1.66–2.35)	
Banerjee *et al*. [48]	*	Prospective study	5912	HR (95% CI)	AF patients: eGFR<30 vs eGFR >60	2.45	4.31 (3.27–5.68)	
Hung *et al*. [49]	Taiwan	Case-control study	11,019	HR (95% CI)	ESRD: AF vs Non-AF	**1.8, 3.3	1.36 (1.147–1.617)	0.0004
Hung *et al*. [49]	Taiwan	Case-control study	11,019	HR (95% CI)	Non-ESRD: AF vs Non-AF	**2.8, 4.4	1.838 (1.538–2.197)	<0.0001

HR, hazard ratio; ESRD, end stage renal disease; AF, atrial fibrillation; CKD, chronic kidney disease; eGFR, estimated glomerular filtration rate. *Data was not identified on article. **Data was reported as median follow-up duration for individual sub-cohorts rather than mean follow-up duration.

The use of oral anticoagulants (OACs) in patients with CKD is controversial and careful consideration must be taken when deciding to start this particular group of patients on OAC. Balancing the risk of bleeding and clotting is a recurring challenge in clinical practice, especially in individuals requiring renal replacement therapy (Table 2 (Ref. [50]). Given that patients with AF and CKD undergo changes in drug pharmacokinetics in addition to a greater propensity for bleeding, the overall net benefit is difficult establish (Table 3 (Ref. [50])). A Serum creatinine concentration >1.5mg/dL has been identified as an independent risk factor for major bleeding events [25]. Furthermore, an eGFR <60 mL/min/1.73 $m^{2}$ has been associated with a significantly increased risk of haemorrhagic stroke [51]. With regard to OAC, the non-vitamin K oral antagonists (NOAC) were considered to be more suitable than vitamin K antagonists (VKA) in view of their favourable pharmacokinetic profile, shorter half-life, and preferable drug interaction profile. In spite of the advantages above, their significant dependency on renal elimination introduces substantial implications and considerations for the overall efficcy and safety profile. A systematic review and meta-analysis conducted by Andò *et al*. [52] reported a better net clinical profile for AF patients with moderate CKD using Apixaban or Edoxaban. The RE-LY, ARISTOTLE, ROCKET-AF, and ENGAGE AF-TIMI 48 randomized controlled trials (RCTs) were landmark RCTs that contributed significantly to the body of evidence surrounding NOAC use in AF stroke prevention [53, 54, 55, 56]. The RCTs conveyed a general trend of non-inferiority of NOAC to warfarin with respect to prevention of stroke and systemic embolization. Certain studies also conveyed a significant reduction in bleeding events in the direct oral anticoagulation (DOAC) cohort [53, 54, 56]. The renal function of all the RCT cohorts was similar with majority at the mild-moderate category of renal impairment (RE-LY: CrCl >50 mL/min – 14,592 (80.5%), ARISTOTLE – CrCl >50 mL/min – 15,161 (83.3%), ROCKET-AF – Median – 67 (52–88) (DOAC group), ENGAGE AF-TIMI – 17,031 (80.7%)). Overall, the RCT findings supported the clinical efficacy and safety of DOAC therapy in AF patients with a significant proportion of the patients in the mild-moderate category of renal impairment.

**Table 2.** **Mechanisms which increase the risk of thrombosis in patients with CKD stage 3–5/ND [50]**.

Mechanisms of thrombosis in patients with CKD stage 3–5/non-dialysis (ND)
↑ Chronic inflammation (endothelial dysfunction)
Hypercoagulability (↑ thrombin antithrombin complex factor VIII and impaired protein C response)
Greater degree of higher coagulability compared to fibrinolysis
Stasis and turbulence of blood flow
Platelet dysfunction

CKD, chronic kidney disease; ND, non-dialysis.

**Table 3.** **Mechanisms which increase the risk of bleeding in patients with CKD stage 5 on dialysis [50]**.

Mechanisms of increased bleeding events in patients with CKD stage 5 on dialysis
↑ Vascular prostaglandin $I_{2}$
↓ von Willebrand Factor
↑ Parathyroid Hormone
↑ Chronic inflammation
↑ Nitric oxide bioavailability
Accumulation of uraemic toxins and Guanidnosuccinic acid
Abnormal platelet adhesion and aggregation

CKD, chronic kidney disease.

The evidence base for OAC in AF patients with severe CKD has been relatively scarce as many of the landmark RCTs that contributed to the evidence, systematically excluded severe CKD [53, 54, 55, 56]. In addition, there was an under-representation of patients in the moderate-to-severe CKD category. A systematic review and meta-analysis of observational studies was conducted by Chokesuwattanaskul *et al*. [57] reported that apixaban was associated with a lower risk of major bleeding events compared to warfarin in patients with advanced CKD and end stage renal disease (ESRD), while risk of thromboembolic events were overall similar. The findings were also consistent with a retrospective matched-cohort study conducted by Siontis *et al*. [58]. The overall uncertainty surrounding OAC in AF and CKD is reiterated by a lack of consensus comparing international guidelines. The Canadian Cardiovascular Society (CCS) 2014 guidelines indicated that VKA can be considered in patients exhibiting an eGFR between 15–30 mL/min that are not established on renal replacement therapy (RRT) while they advise against OAC for ESRD [59]. The American College of Cardiology/American Heart Association/Heart Rhythm Society (ACC/AHA/HRS) guidelines advocated for NOAC usage in CrCl down to 15 mL/min and VKA prescription irrespective of renal function or RRT status [60]. The European Society of Cardiology (ESC) indicate that OAC can be prescribed safely in CrCl >15 mL/min, however, do not provide clear consensus in ESRD [61]. The CHEST 2018 guidelines synthesised by the American College of Chest Physicians, advocated for an individualised decision-making process along with meticulous VKA administration for time-in-therapeutic range above 65–70% in patients with ESRD [62].

To conclude, at present, the evidence-based recommendations for anticoagulation in patients with AF and CKD indicate that there is a benefit in those with CKD stage 2–3 and there is consensus of net benefit for select patients with CKD stage 4, however, in patients with CKD stage 5 there is uncertainty and likely net harm and in these cases the decision to commence OAC should be taken on a case by case basis.

## 5. Arrhythmia Management in CKD

Patients with CKD or ESKD are often excluded from trials studying rate vs rhythm control and therefore there is a lack of evidence on how to manage AF in patients with CKD [63]. The decision to pursue a rate or rhythm control strategy in patients with CKD depends on their individual characteristics such as co-morbidities, duration of AF, symptom severity, contraindications to the use of anti-arrhythmic drugs (AADs) and the patient’s personal preference [64]. Overall the indications and considerations for a rhythm control strategy in CKD patients is similar to the general populations. There is a paucity of RCTs that have evaluated specific anti-arrhythmic strategies of rate vs. rhythm control in patients with CKD or ESRD. A post hoc analysis of the Global Utilization of Streptokinase and t-PA for Occluded Coronary Arteries (GUSTO) III trial reported that neither rate or rhythm control strategy significantly impacted short term or long-term mortality, irrespective of renal function.

The common drug classes administered for rate control include beta-blockers, non-dihydropyridine calcium channel blockers, and digoxin [65]. Water-soluble pharmacological agents are prone to accumulation in CKD due to impaired renal elimination and thus water-soluble beta-blocker therapies such as atenolol should typically be avoided [65]. Bisoprolol exhibits a mixed metabolism profile that may require dose adjustment based on the degree of renal impairment. Carvedilol is a lipophilic beta-blocker and exhibits minimal renal elimination with dose adjustments not considered to be required for CKD [66]. Digoxin is typically avoided in severe CKD as majority of the agent undergoes renal elimination [67]. The usage in CKD is complicated by a narrow therapeutic index, long half-life, and predisposition to arrhythmogenesis in the presence of abnormalities such as hypokalaemia which can occur during dialysis [68]. Yang *et al*. [67] conducted a population-based cohort study and reported an association of increased mortality with CKD. Although non-dihydropyridine calcium channel blockers such as Diltiazem and Verapamil can be used, these should be avoided in patients with left ventricular systolic dysfunction [12].

Rhythm control maybe the favoured option in patients where rate control is difficult to achieve, the patient is young or there is evidence of tachycardia mediated cardiomyopathy. There are several AADs which can be used in patients with CKD, however, they must be prescribed with caution in view of renal clearance as well as proarrhythmic risks in patients with structural heart disease (see Table 4 (Ref. [69, 70, 71])). Amiodarone is among the most common anti-arrhythmic agents used to treat AF and is neither eliminated through the renal system or dialyzable. A large data set retrospective study conducted by Ullal *et al*. [72] conveyed that amiodarone does not negatively affect survival in patients with ESRD. The propensity for adverse events/organ toxicity secondary to amiodarone in patients with CKD is currently yet to be established. A prospective, nationwide registry of AF patients reported that Amiodarone was the most commonly prescribed anti-arrhythmic in stage IV/V CKD (68.6%, *p* < 0.0001) [73]. Flecainide undergoes both liver metabolism and renal elimination, requires dose reduction if eGFR <35 mL/min/1.73 $m^{2}$ and caution is pertinent especially in consideration of structural heart disease [69, 74]. Sotalol is predominantly excreted through the kidneys and is dialyzable as well as pro-arrhythmic in CKD patients, as such caution in renal impairment is highly advised [64].

**Table 4.** **Common AADs used in management of AF, metabolism and excretion and cautions that the prescriber should be aware of**.

Anti-arrhythmic drug	Metabolism/clearance	Caution
Propafenone	Liver metabolism/Renal excretion	Avoid in patients with heart failure & significant left ventricular hypertrophy (LVH).
Sotalol	Not metabolised/renally excreted	Pro-arrhythmic in CKD, hypomagnesia, hypokalaemia. Increased risk of Torsades de pontes (TdP) in patients on dialysis [70].
		Dialyzable – administer maintenance dose after dialysis.
Amiodarone	Liver metabolism/Biliary excretion	Thyroid dysfunction, pulmonary toxicity – even at low doses.
Flecanide	Minimal liver metabolism/renal excretion	Avoid in patients with severe CKD due to increased risk of toxicity [71].
		Avoid in patients with significant structural heart disease [69].

AADs, antiarrhythmic drugs; CKD, chronic kidney disease; AF, atrial fibrillation.

A rhythm control strategy using direct current cardioversion (DCCV) has limited and inconsistent evidence. One study assessing patients with CKD and post-myocardial infarction AF concluded that 70% of patients with CKD and managed with DCCV were discharged in sinus rhythm, compared to 84% who had preserved renal function [75]. Schmidt *et al*. [76] also observed that patients with AF and moderately or severely impaired renal function were more likely to have recurrence. In contrast to previously mentioned studies, Reinecke *et al*. [7] reported findings from a large nationwide prospective registry and indicated that the success rate of restored sinus rhythm was very similar with 79.5–82.9% of patients successfully treated with DCCV irrespective of their renal function. In addition, Schmidt *et al*. [76] reported that patients with moderate renal impairment showed an increase in eGFR where sinus rhythm was maintained for 1 month post DCCV. Although DCCV may be considered for highly symptomatic or relatively recent onset AF, it is typically insufficient to maintain normal sinus rhythm in patients with long standing persistent AF, permanent AF and/or severe LA dilation, thus long term anti-arrhythmic medications, catheter ablation or a pace and ablate strategy may be considered depending on the patients’ symptoms and/or the presence heart failure.

## 6. Catheter Ablation in CKD

Although catheter ablation is a well-established management option for rhythm control in AF, the evidence base and effect of CKD on outcomes in patients with CKD who have an ablation is limited. There are several predictors of recurrence of AF in patients undergoing catheter ablation (CA) such as enlarged left atrium (LA) and persistent AF [77]. Several studies have analysed the impact of impaired renal function on CA*.* Chao *et al*. [78] looked at 232 patients who underwent CA and concluded that in patients with PAF, a reduced eGFR was associated with a higher recurrence rate. Naruse *et al*. [79] studied 221 patients with CKD (defined as an eGFR <60 mL/min/1.73 $m^{2}$) and AF who underwent CA. On following up these patients over a mean period of 32 months it was found that patients with CKD had a higher recurrence rate compared to patients without CKD (57.4% vs 33.5%, *p* < 0.01). However, patients with CKD were of older age with greater left atrial volumes and more likely to have hypertension. These multiple factors can make it difficult to attribute AF recurrence to CKD alone [79]. Sairaku *et al*. [80] carried out a study with a smaller group of patients receiving maintenance haemodialysis who underwent CA for AF and concluded that when compared with age-sex matched patients who did not have ESKD, recurrence rates were higher. A systematic review and meta-analysis of 4 studies evaluating the efficacy of catheter ablation in CKD, conveyed a higher AF recurrence risk following single catheter ablation [81]. With respect to cryo-ablation, Yanagisawa *et al*. [82] reported that renal impairment at baseline was an independent predictor of recurrence and also observed a significant prevalence of non-pulmonary vein ectopic beats in patients with CKD. In contrast, Takahashi *et al*. [83] reported that successful treatment of AF by CA was associated with an improvement in renal function at 1 year follow-up in patients with mild-moderate renal impairment.

Overall, it appears that the evidence base for catheter ablation in patients with AF and CKD is limited. While there appears to be potentially some benefit when successfully conducted, recurrence rates especially in increasing severities of CKD, are significant and considerable. As a result, further robust evaluation into outcomes corresponding to specific CKD patient groups might be beneficial to optimise patient selection.

## 7. Conclusions

Managing patients with concomitant AF and CKD is complex. The limited evidence base for managing these patients can present a challenge to the physician when considering management options. There is a close cyclical relationship between AF and CKD and the progression of both diseases [84]. As stated in the ESC guidelines a shared decision-making process is required between the physician and the patient [61]. This pertains to all aspects of AF management in patients with CKD, including weighing up the risks and benefits of OAC, pursuing a rate or rhythm control strategy and deciding upon CA where there is likely to be of clinical benefit.
